# Trait Anxiety and Cyberbullying Among University Students: The Mediating Role of Intolerance of Uncertainty and the Moderating Effect of Mindfulness

**DOI:** 10.3390/bs16101901

**Published:** 2026-10-14

**Authors:** Yueyue Zhu, Shuangxi Zhang, Pengyun Yan, Bing Qi

**Affiliations:** 1 College of Education, Hebei University, Baoding 071051, China; 20248004031@stumail.hbu.edu.cn (Y.Z.); 20249004011@stumail.hbu.edu.cn (S.Z.); 2 Shijiazhuang Prison, Shijiazhuang 050011, China; y1632293628@163.com

**Keywords:** trait anxiety, cyberbullying, intolerance of uncertainty, mindfulness, moderated mediation

## Abstract

Cyberbullying threatens psychological well-being and the quality of online social environments. This study examined the statistical association of trait anxiety with cyberbullying, with intolerance of uncertainty as a statistical intermediary, utilizing the General Aggression Model and Monitoring and Acceptance Theory, and investigated whether mindfulness moderates this association. A cross-sectional study was performed, including 1247 university students. Findings from Hayes’ PROCESS Model 8 indicated that trait anxiety is significantly and positively related to cyberbullying, with intolerance of uncertainty serving as a statistical intermediary in this relationship. Moreover, mindfulness significantly moderated both the first-stage pathway and the direct association between trait anxiety and cyberbullying. The positive association of trait anxiety with intolerance of uncertainty and cyberbullying was considerably diminished by elevated levels of mindfulness. These findings identify intolerance of uncertainty as a cognitive-affective statistical indirect pathway linking anxiety-related vulnerability to cyberbullying and suggest that mindfulness may serve as a potential protective correlate. They also point to possible targets for future longitudinal and intervention research.

## 1. Introduction

The rapid expansion of social media has made cyberbullying a persistent problem in digital environments (Kasturiratna et al., 2025; Luo et al., 2025). Cyberbullying refers to intentional and repeated aggressive behavior conducted through electronic media with the aim of harming, humiliating, or intimidating others (Slonje et al., 2013). Accumulating evidence shows that cyberbullying is associated with adverse psychological outcomes, including anxiety, depression, emotional distress, and impaired functioning in online communities (Aparisi et al., 2025; Monteiro et al., 2024; Song et al., 2024). Although prior studies have increasingly examined situational stressors and cybervictimization as antecedents of online aggression (R. Chen et al., 2025), less is known about how stable anxiety-related dispositions are statistically associated with cyberbullying behavior. University students are a particularly relevant population because they are intensive users of digital communication technologies and often experience academic, interpersonal, and developmental pressures. The present study therefore examined the psychological pathway through which trait anxiety may be statistically associated with cyberbullying among students across different undergraduate years.

The General Aggression Model (GAM) provides a useful framework for explaining how person-related vulnerabilities and situational inputs jointly shape aggressive behavior (Allen et al., 2018). According to the GAM, anxiety-related arousal and threat appraisal may be statistically associated with affective states, cognition, and behavioral scripts and thereby with a higher likelihood of aggressive responses (Bloomfield et al., 2024). Within this framework, intolerance of uncertainty can be understood as a specific cognitive–affective appraisal process that amplifies anxiety-related arousal: when individuals perceive uncertainty as threatening, they experience heightened distress and a strong desire for cognitive closure, which may be statistically associated with aggressive behavioral scripts as a maladaptive means of restoring perceived control. Importantly, although trait anxiety is classically conceptualized as an internalizing disposition associated with withdrawal and social avoidance, it may also predispose individuals to reactive aggression under conditions of perceived threat (Cheng et al., 2025; Endler & Kocovski, 2001). Individuals with high trait anxiety tend to be more vigilant to potential threats and more likely to interpret ambiguous interpersonal cues as negative or hostile (Cheng et al., 2025; Endler & Kocovski, 2001). This heightened vigilance is theoretically linked to intolerance of uncertainty through a hostile attribution bias and a desire for cognitive closure—anxious individuals perceive ambiguous social cues as threatening and struggle to tolerate the associated uncertainty, which in turn predisposes them toward maladaptive aggressive responses. In online settings, where cues are often incomplete and interactions can be rapid, anonymous, or emotionally charged (Slonje et al., 2013; H. Chen et al., 2024), this heightened threat sensitivity—combined with a desire to reduce uncertainty and restore a sense of control (Birrell et al., 2011; Wilson et al., 2026)—may increase the risk of cyberbullying as a maladaptive response to perceived social threat or emotional discomfort (Clauss et al., 2026; Guo et al., 2025). Monitoring and Acceptance Theory (MAT) complements this GAM-based account by explaining how mindfulness may buffer these processes. According to MAT, mindfulness involves two complementary components: monitoring present-moment experiences and accepting those experiences without excessive avoidance or reactivity (Simione & Saldarini, 2023). The monitoring component corresponds to heightened interoceptive and environmental awareness that may initially increase sensitivity to distress, whereas the acceptance component directly counteracts GAM’s aggressive action tendency by promoting non-reactivity and cognitive decoupling. Through this acceptance process, individuals can observe anxious thoughts and impulses without immediately acting on them, thereby weakening the link between anxious arousal and cyberbullying behavior. Furthermore, some students may occupy both victim and perpetrator roles over time; for those with high trait anxiety, prior victimization may sensitize threat appraisal and increase the likelihood of reactive perpetration, forming a cycle of victim-perpetrator overlap (Guo et al., 2025). Thus, while anxiety is commonly linked to victimization, under the conditions of digital communication—where ambiguity is high and immediate accountability is low—it may also be associated with perpetration.

**H1.**  *Trait anxiety is positively associated with cyberbullying among university students.*

Intolerance of uncertainty (IU) may help explain why trait anxiety is linked to cyberbullying. IU refers to a dispositional difficulty in enduring uncertain or ambiguous situations and is sustained by the belief that uncertainty is threatening or unacceptable (Birrell et al., 2011). Individuals with higher trait anxiety are more likely to evaluate unclear situations as dangerous and to experience stronger distress when outcomes are unpredictable (Raugh et al., 2025). Recent work has further identified IU as a transdiagnostic risk factor across anxiety-related problems (Wilson et al., 2026; Çorak, 2026). In digital communication, ambiguous messages, delayed responses, and unclear social feedback may intensify uncertainty-related distress (H. Chen et al., 2024). When individuals lack adequate regulatory resources, cyberbullying may become a maladaptive attempt to restore perceived control, discharge negative emotion, or reduce interpersonal uncertainty (Clauss et al., 2026). Therefore, IU may function as a hypothesized cognitive-affective mechanism linking trait anxiety to cyberbullying.

**H2.**  *Intolerance of uncertainty serves as a statistical intermediary in the association between trait anxiety and cyberbullying.*

Mindfulness may weaken the risk pathway from trait anxiety to cyberbullying. Mindfulness is commonly defined as present-centered awareness of bodily sensations, emotions, thoughts, and environmental events, accompanied by an accepting and nonjudgmental attitude (Chems-Maarif et al., 2025). Although rooted in Buddhist contemplative traditions, mindfulness has been widely adapted in psychological research and intervention contexts (Cook & Cassaniti, 2022; Prakash et al., 2025). Monitoring and Acceptance Theory (MAT) proposes that mindfulness involves two complementary components: monitoring present-moment experiences and accepting those experiences without excessive avoidance or reactivity (Simione & Saldarini, 2023). Monitoring may increase sensitivity to internal states, whereas acceptance helps reduce emotional reactivity and maladaptive behavioral responses (Raugh et al., 2025; Simione & Saldarini, 2023).

A growing body of evidence suggests that mindfulness is associated with stronger self-regulation, greater cognitive flexibility, and more adaptive emotion regulation (Bais, 2026; Kong et al., 2025; Ma et al., 2025; Pang et al., 2025). These capacities may help anxious individuals observe distressing thoughts and feelings without immediately acting on them (Durham et al., 2025). In the context of cyberbullying, mindfulness may therefore reduce the direct translation of trait anxiety into cyberbullying perpetration (Zhang, 2025). At the same time, mindfulness may also weaken the association between trait anxiety and IU by allowing individuals to tolerate ambiguity more effectively and remain oriented toward the present moment rather than engaging in threat-oriented anticipation (Beylin & Zawadzki, 2026; Gan et al., 2026). Accordingly, the present study tested whether mindfulness moderates both the direct path from trait anxiety to cyberbullying and the first stage of the indirect path from trait anxiety to IU.

**H3.**  *Mindfulness moderates the association between trait anxiety and cyberbullying, such that the positive association is weaker among students with higher levels of mindfulness.*

**H4.**  *Mindfulness moderates the association between trait anxiety and intolerance of uncertainty, such that the positive association is weaker among students with higher levels of mindfulness.*

**Figure 1:**
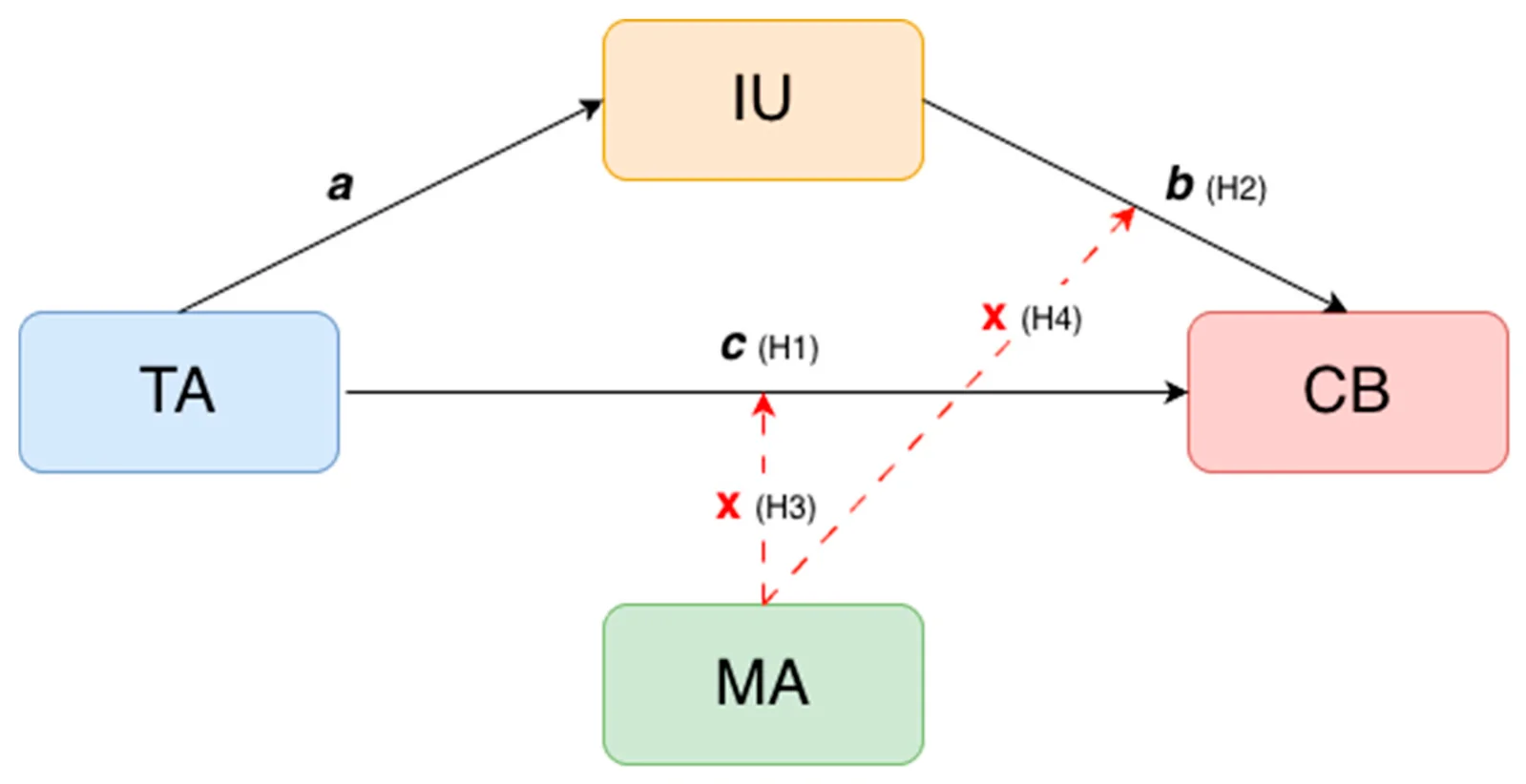
Hypothesized moderated mediation model. TA = trait anxiety; IU = intolerance of uncertainty; CB = cyberbullying perpetration; MA = mindfulness. Solid lines represent direct and indirect pathways; dashed lines represent moderating effects. “×” indicates the interaction term (Trait Anxiety × Mindfulness) for the respective moderated path. H1–H4 indicate the corresponding hypotheses.

Taken together, this study tested a moderated mediation model in which trait anxiety is associated with cyberbullying both directly and indirectly through IU, and mindfulness buffers both the first-stage pathway and the direct pathway (Figure 1). This model extends prior work by integrating an anxiety-related vulnerability, an uncertainty-related cognitive process, and a mindfulness-based regulatory factor within a single framework.

## 2. Materials and Methods

### 2.1. Participants

A convenience sampling strategy was used to recruit university students from one university in Hebei Province, China, through an online questionnaire survey (Wenjuanxing, Ranxing Information Technology Co., Ltd., Beijing, China). Data collection took place from January 2026 to June 2026. Students were recruited through their academic departments and class groups via class advisors and group chat platforms (WeChat, Tencent Holdings Ltd., Shenzhen, Guangdong, China). Inclusion criteria were current enrolment as an undergraduate student and willingness to provide informed consent; exclusion criteria included inability to complete the survey independently and failure to pass attention-check items. No financial or course credit incentives were offered. The online platform restricted submissions by IP address to prevent duplicate responses. Two embedded attention-check items (e.g., “Please select ‘Agree’ for this question”) were included. “Patterned responses” were operationally defined as identical ratings across consecutive items (>90% similarity). “Logical inconsistencies” were defined as contradictory responses to reverse-scored item pairs. A pilot study (*n* = 50) was conducted prior to data collection, with a mean completion time of 320 s (SD = 27 s); the 240 s threshold was established as the pilot mean minus three standard deviations and was applied before analysis of the final sample. The complete questionnaire included 85 scale items plus demographic questions, totalling 92 items. A total of 1368 questionnaires were initially collected. Data were screened using the following criteria: (a) 52 questionnaires were excluded because of implausibly short completion times (<240 s), a threshold determined as the pilot-study mean completion time minus three standard deviations; (b) 41 questionnaires were excluded because of patterned responses or failed attention check items; and (c) 28 questionnaires were excluded because of incomplete responses or logical inconsistencies. The final sample included 1247 valid questionnaires, yielding a valid data retention rate of 91.15%. The valid sample consisted of 586 male students (47.0%) and 661 female students (53.0%). Participants ranged in age from 17 to 25 years (M = 20.43, SD = 1.59). All participants provided online informed consent prior to participation. Because the sample included participants aged 17, a parental/guardian consent waiver was approved by the Ethics Committee of Hebei University Affiliated Hospital (protocol code HDFYLL-KY-2024-090). This waiver was granted on the basis that (a) the research involved no more than minimal risk (an anonymous questionnaire survey with no sensitive personal data or intervention), (b) obtaining parental permission would not be practical due to the anonymous survey format, and (c) participants were university students aged 17 or older, who were considered capable of independent decision-making under the institutional ethical framework for minimal-risk research. By academic year, 312 participants were first-year students (25.0%), 349 were second-year students (28.0%), 324 were third-year students (26.0%), and 262 were fourth-year students or above. Regarding daily internet use and previous cybervictimization, these were also assessed (Bansal et al., 2025; Soriano-Molina et al., 2025). Regarding daily internet use, 19.4% of participants reported using the internet for less than 3 h per day, 31.1% for 3–6 h per day, and 49.4% for more than 6 h per day. In addition, 436 participants (35.0%) reported previous cybervictimization. These demographic and background variables were included as covariates based on theoretical and empirical considerations: gender, age, major, academic year, and daily Internet use have been shown in prior research to be associated with cyberbullying involvement (Bansal et al., 2025; Soriano-Molina et al., 2025); previous cybervictimization was included to account for its potential confounding role in the anxiety–perpetration relationship. We acknowledge that cybervictimization may also serve other functions (e.g., moderator, antecedent, reciprocal outcome, or part of a bully-victim process), and distinguishing these roles is an important direction for future research.

### 2.2. Measures

Internal consistency for all scales was assessed using both Cronbach’s alpha and McDonald’s omega. Cronbach’s alpha was based on Pearson correlations. McDonald’s omega total (ω_t_) was estimated using the polychoric correlation matrix, which is appropriate for ordinal Likert-type items, via the psych package (Version 2.3.6) in R (Version 4.3.1).

#### 2.2.1. Trait Anxiety

Trait anxiety was measured using the Trait Anxiety subscale of the State-Trait Anxiety Inventory Form Y (STAI-Form Y) (Spielberger, 1983). The full inventory contains 40 items across two subscales: State Anxiety and Trait Anxiety. Because the present study focused on stable anxiety proneness, only the 20-item Trait Anxiety subscale was used. Participants responded on a 4-point scale ranging from 1 (almost never) to 4 (almost always). Ten items were reverse scored. A sample item is “I worry too much over something that really doesn’t matter.” Higher total scores indicate higher trait anxiety. In the present sample, the scale demonstrated good internal consistency (Cronbach’s α = 0.811; McDonald’s ω = 0.836).

#### 2.2.2. Revised Cyberbullying Inventory

Cyberbullying perpetration was measured using the Cyberbullying Perpetration subscale of the Revised Cyberbullying Inventory (RCBI) (Chu & Fan, 2017), which was developed based on the Cyberbullying Inventory (Topçu & Erdur-Baker, 2010). The full scale contains 28 items across two subscales: Cyberbullying Perpetration and Cyberbullying Victimization. Because the present study focused on cyberbullying perpetration, only the 14-item Cyberbullying Perpetration subscale was used. Participants rated how often they had engaged in each behavior during the past six months on a 4-point scale ranging from 1 (never) to 4 (more than three times). A sample item is “Sent threatening or hurtful text messages to someone.” Higher scores indicate greater involvement in cyberbullying perpetration. In the present sample, the scale demonstrated excellent internal consistency (Cronbach’s α = 0.941; McDonald’s ω = 0.958).

#### 2.2.3. Intolerance of Uncertainty (IU)

Intolerance of uncertainty was assessed using the 12-item Intolerance of Uncertainty Scale (IUS-12) (Carleton et al., 2007). Participants rated each item on a 5-point scale ranging from 1 (not at all characteristic of me) to 5 (entirely characteristic of me). A sample item is “I can’t stand being taken by surprise.” Higher total scores indicate greater intolerance of uncertainty. In the present sample, the scale demonstrated good internal consistency (Cronbach’s α = 0.900; McDonald’s ω = 0.914).

#### 2.2.4. Mindfulness

Mindfulness was assessed using the Chinese version of the Five Facet Mindfulness Questionnaire (Ch-FFMQ) (Deng et al., 2011). The scale includes 39 items across five facets: observing, describing, acting with awareness, nonjudging, and nonreacting. Participants rated items on a 5-point Likert scale ranging from 1 (not at all true) to 5 (completely true). A sample item is “I perceive my feelings and emotions without having to react to them.” Higher total scores indicate higher levels of mindfulness. In the present study, the total FFMQ score was used as the primary indicator of dispositional mindfulness. This decision was based on the conceptual rationale that the FFMQ total reflects a general tendency toward present-moment awareness and nonreactive acceptance—both of which align with the integrated monitoring and acceptance functions proposed by MAT (Simione & Saldarini, 2023). Specifically, the Observing and Describing facets primarily capture the monitoring component, while Acting with Awareness, Nonjudging, and Nonreacting primarily capture the acceptance component, with the total score representing the combined operation of these processes.

Confirmatory factor analyses (CFA) were conducted to verify the factor structure of each scale in the present sample using the lavaan package (Version 0.6-21) in R (Version 4.4.3). Because all items were measured on ordinal Likert scales (4-point or 5-point), the WLSMV (Weighted Least Squares Mean and Variance adjusted) estimator was used, which is appropriate for categorical/ordinal data. No item parceling was used; all analyses were conducted at the item level. No residual correlations were freely estimated. For the STAI-T, RCBI, and IUS-12, unidimensional (single-factor) models were estimated based on their theoretical foundations (Table 1). The results indicated good fit across all three scales (STAI-T: χ^2^ = 182.75, df = 170, CFI = 0.997, TLI = 0.997, RMSEA = 0.008 [90% CI: 0.000, 0.015], SRMR = 0.025; IUS-12: χ^2^ = 65.04, df = 54, CFI = 0.999, TLI = 0.999, RMSEA = 0.013 [90% CI: 0.000, 0.023], SRMR = 0.015; RCBI: χ^2^ = 78.02, df = 77, CFI = 1.000, TLI = 1.000, RMSEA = 0.003 [90% CI: 0.000, 0.016], SRMR = 0.014). Standardized factor loadings ranged from 0.412 to 0.490 for STAI-T, 0.755 to 0.821 for RCBI, and 0.645 to 0.709 for IUS-12 (all *p*s < 0.001), supporting the unidimensionality of these scales.

For the FFMQ, we tested five specifications: unidimensional, five-correlated-factor, higher-order, bifactor, and ESEM (see Supplementary Materials Tables S1 and S2 for full syntax and output). The five-correlated-factor model converged but yielded latent correlations ranging from 0.995 to 1.028, with 8 of 10 correlations at or above 1.00. Following the reviewer’s recommendation, we explicitly interpret this pattern as an improper solution rather than merely severe multicollinearity. The higher-order and ESEM models did not converge due to non-positive definite latent covariance matrices, consistent with the improper five-factor solution.

To examine whether this improper solution reflected a scoring, coding, or correlation-matrix artifact, we conducted a series of diagnostic checks. First, all 39 items were verified against the scoring manual of the Chinese version of the FFMQ (Deng et al., 2011); the 19 reverse-scored items were correctly reverse-scored, and all item–total correlations were positive (range = 0.503 to 0.580). Second, all items were within the intended 1–5 range with no missing data. Third, the 741 inter-item polychoric correlations ranged from 0.234 to 0.381 (median = 0.308; maximum pair = MF19–MF25, r = 0.381), indicating that the improper solution was not driven by problematic polychoric correlations. Fourth, standardized CFA loadings ranged from 0.505 to 0.591 (all *p*s < 0.001), showing that individual items performed well at the item level. Fifth, an exploratory factor analysis using polychoric correlations with promax rotation and minimum residual extraction showed that only 20 of 39 items loaded ≥ 0.30 on any factor, and the five factors together explained only 33.6% of the total variance; the extracted factors did not align with the theoretical five-facet structure. Sixth, discriminant validity indices were uniformly inadequate: HTMT values ranged from 0.995 to 1.027 (8 of 10 ≥ 1.00), and AVE values ranged from 0.291 to 0.320, well below the recommended threshold of 0.50. Finally, modification indices revealed substantial local dependence involving MF15 (MI = 251), MF1 (MI = 247), and MF25 (MI = 237), which is consistent with the improper five-factor solution. Taken together, these convergent diagnostics indicate that the five FFMQ facets were not empirically distinguishable in this sample.

We therefore used the FFMQ total score only as a pragmatic manifest composite in the main structural model. This decision does not imply that the FFMQ is psychometrically unidimensional, nor does it establish discriminant validity among the five facets. The total scale demonstrated excellent internal consistency (α = 0.937; ω = 0.945), and the five facets showed acceptable reliability (α ranging from 0.739 to 0.755). All CFA syntax, diagnostic results, and alternative model specifications are reported in Supplementary Materials Tables S1 and S2.

### 2.3. Data Analysis

Preliminary analyses included descriptive statistics, independent-samples *t* tests, one-way analyses of variance, and bivariate correlations. The moderated mediation model was tested using Hayes’ PROCESS macro (Model 8). In this model, trait anxiety was specified as the independent variable, cyberbullying as the dependent variable, intolerance of uncertainty as the mediator, and mindfulness as the moderator of both the path from trait anxiety to intolerance of uncertainty and the direct path from trait anxiety to cyberbullying. Gender, age, academic year, major, daily internet use, and previous cybervictimization were included as covariates. The analysis was conducted using SPSS Statistics (Version 27.0) with the PROCESS macro (Version 4.2; Hayes, 2022). Conditional indirect effects were evaluated using bootstrapped confidence intervals with 5000 resamples and bias-corrected 95% confidence intervals; effects were considered significant when the 95% confidence interval did not include zero. No missing data were present after screening, so listwise deletion was not required. All continuous predictors (trait anxiety and mindfulness) were mean-centered prior to analysis. Categorical covariates were dummy-coded as follows: gender (0 = male, 1 = female), major (0 = liberal arts, 1 = science/engineering), and cybervictimization experience (0 = no, 1 = yes). Daily internet use was coded ordinally (1 = <3 h, 2 = 3–6 h, 3 = >6 h) and entered as a continuous covariate. Academic year was coded 1 = first-year, 2 = second-year, 3 = third-year, 4 = fourth-year or above and treated as continuous in the main model. A sensitivity analysis using dummy-coded academic year (with first-year as the reference category) yielded materially identical results (Supplementary Table S3). Significant interactions were probed using simple slopes at low (−1 SD), mean, and high (+1 SD) levels of mindfulness. To justify the statistical power of the present sample, a post hoc sensitivity power analysis was conducted using G*Power version 3.1.9.7 (Faul et al., 2009). For linear multiple regression with nine predictors, an alpha of 0.05, and power of 0.80, the minimum detectable effect size (f^2^) was approximately 0.012. The observed effect sizes for the two interaction terms—calculated using the R^2^-change method (Cohen, 1988)—were f^2^ = 0.0229 for the interaction predicting intolerance of uncertainty and f^2^ = 0.0256 for the interaction predicting cyberbullying, both exceeding the minimum detectable threshold and meeting the conventional criterion for a small effect (f^2^ = 0.02). Simulation-based approaches for power analysis of conditional process models suggest that bootstrap procedures with 5000 resamples provide reliable confidence interval estimation for moderated mediation effects when sample sizes are adequate (Xu et al., 2024; Preacher et al., 2007).

## 3. Results

### 3.1. Tests of Demographic Differences on Key Variables

Independent-samples *t* tests were conducted to examine gender differences in the main study variables. No significant gender difference was found for cyberbullying, *t*(1211.70) = 1.41, *p* = 0.16. Trait anxiety did not reach the conventional significance threshold for gender differences, *t*(1245) = 1.91, *p* = 0.06, with male students (*M* = 45.47) scoring slightly higher than female students (*M* = 44.60). No significant gender differences were found for intolerance of uncertainty, *t*(1245) = 0.65, *p* = 0.52, or mindfulness, *t*(1245) = −0.94, *p* = 0.35.

Independent-samples *t* tests comparing liberal arts students with science and engineering students showed no significant differences in trait anxiety, *t*(1245) = −1.28, *p* = 0.20; intolerance of uncertainty, *t*(1245) = −1.24, *p* = 0.22; mindfulness, *t*(1245) = 0.07, *p* = 0.94; or cyberbullying, *t*(1245) = −1.17, *p* = 0.24.

One-way analyses of variance were used to examine differences by academic year and daily internet use. Academic year was not significantly associated with trait anxiety, *F*(3, 1243) = 0.94, *p* = 0.42, or cyberbullying, *F*(3, 1243) = 0.58, *p* = 0.63. Daily internet use was also not significantly associated with cyberbullying, *F*(2, 1244) = 0.02, *p* = 0.98, or trait anxiety, *F*(2, 1244) = 0.77, *p* = 0.46.

Finally, participants with and without previous cybervictimization did not differ significantly in cyberbullying, *t*(1245) = 1.05, *p* = 0.30. However, intolerance of uncertainty showed a marginal difference, *t*(1245) = 1.88, *p* = 0.06, with participants without previous cybervictimization (*M* = 36.32) scoring slightly higher than those with previous cybervictimization (*M* = 35.33). Overall, no statistically significant differences were observed across demographic and background groups on the main variables. However, non-significance does not establish equivalence, and these variables were retained as covariates in subsequent analyses to control for their potential effects. All demographic and background variables were retained as covariates in the subsequent conditional process analyses to control for their potential confounding effects.

### 3.2. Descriptive Statistics and Correlation

Descriptive statistics and bivariate correlations are presented in Table 2. Trait anxiety was positively correlated with intolerance of uncertainty (*r* = 0.38, *p* < 0.001) and cyberbullying (*r* = 0.42, *p* < 0.001), and negatively correlated with mindfulness (*r* = −0.25, *p* < 0.001).

Intolerance of uncertainty was positively correlated with cyberbullying (*r* = 0.39, *p* < 0.001) and negatively correlated with mindfulness (*r* = −0.22, *p* < 0.001). Mindfulness was negatively correlated with cyberbullying (*r* = −0.29, *p* < 0.001). These correlations were consistent with the hypothesized directions and supported further tests of mediation and moderation.

To assess the potential influence of common method variance, a Harman’s single-factor test was conducted. The first unrotated factor accounted for 16.85% of the total variance. While this did not indicate a dominant single factor, this procedure has limited diagnostic capacity and does not exclude common-method variance (Podsakoff et al., 2003; Podsakoff et al., 2012).

The cyberbullying perpetration measure showed a mean of 29.57 (*SD* = 8.25, range: 14–55), with skewness = 0.34 (*SE* = 0.07) and kurtosis = −0.38 (*SE* = 0.14), indicating an approximately normal distribution. The proportion of respondents reporting the minimum possible score (14) was 0.3% (*n* = 4), indicating that floor effects were minimal. Regression diagnostics confirmed that OLS assumptions were sufficiently satisfied. Residuals were approximately normally distributed (Shapiro–Wilk test, W = 0.9935; although the *p*-value was significant due to the large sample size, the W statistic very close to 1 indicates minimal deviation), with no substantial heteroscedasticity (Breusch–Pagan test, *BP* = 10.15, *df* = 10, *p* = 0.428) or multicollinearity (VIFs ranging from 1.00 to 1.23). No influential cases exceeded Cook’s D > 1.0 (maximum Cook’s D = 0.015). The bootstrap method (with 5000 resamples) used for estimating conditional indirect effects further supports the robustness of our findings. These results collectively indicate that OLS regression assumptions are sufficiently satisfied for the present data.

### 3.3. Moderated Mediation Analysis

A conditional process analysis was conducted using Hayes’ PROCESS macro (Model 8) to test whether intolerance of uncertainty mediated the association between trait anxiety and cyberbullying and whether mindfulness moderated the first-stage path and the direct path. Gender, age, academic year, major, daily internet use, and previous cybervictimization were included as covariates (see Table 3).

The total effect of trait anxiety on cyberbullying was significant, *β* = 0.425, *p* < 0.001. After including the mediator and moderator, the direct effect of trait anxiety on cyberbullying remained significant, and intolerance of uncertainty emerged as a significant predictor of cyberbullying, *β* = 0.23, *p* < 0.001. Moderation analysis indicated that the interaction between trait anxiety and mindfulness significantly predicted intolerance of uncertainty (*B* = −0.007, *SE* = 0.001, *β* = −0.137, *t* = −5.33, *p* < 0.001), and the direct path was also significantly moderated by mindfulness (*β* = −0.13, *p* < 0.001).

**Figure 2:**
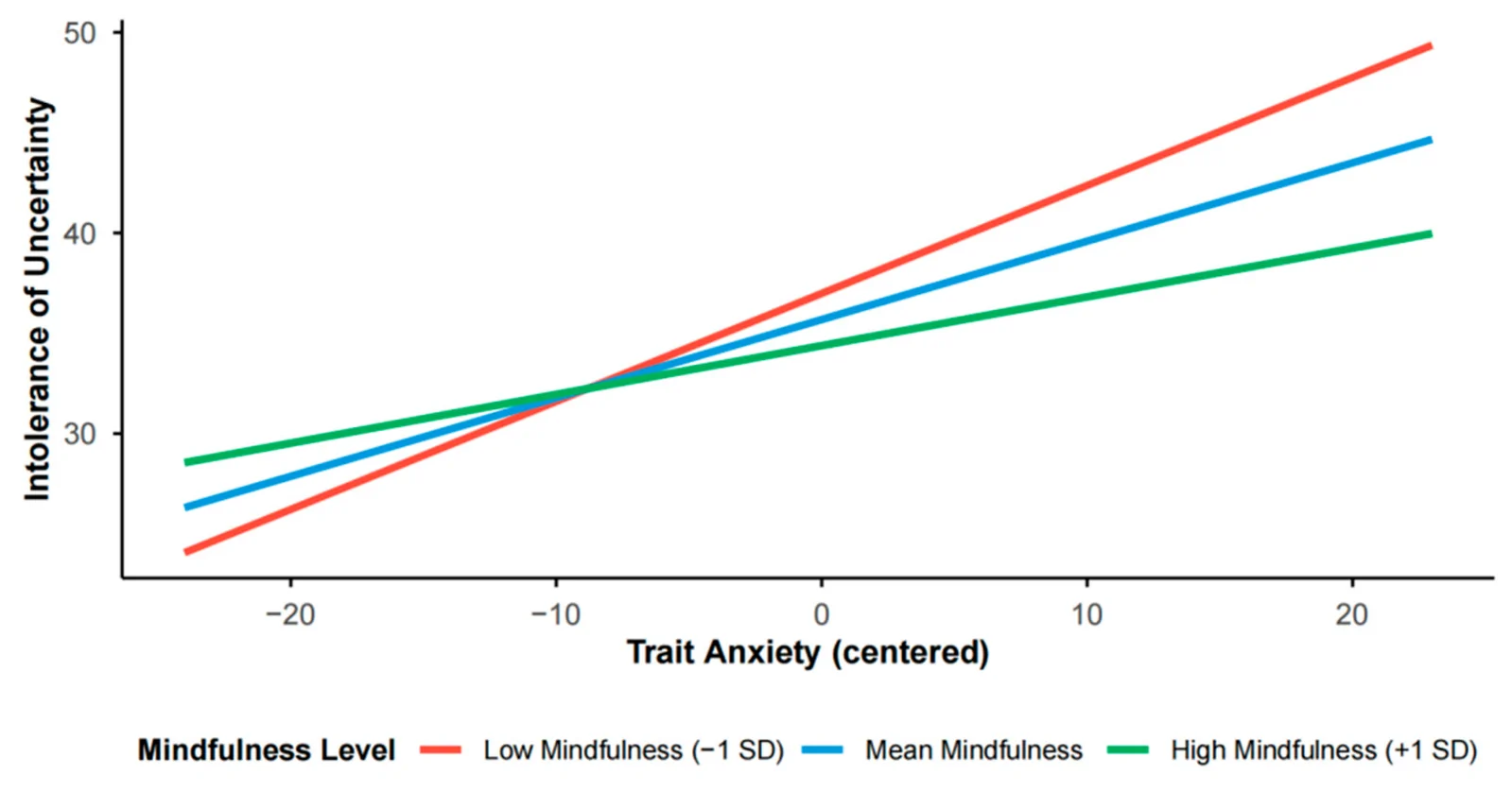
Interaction between trait anxiety and mindfulness on intolerance of uncertainty. Simple slopes are plotted at low (−1 *SD*), mean, and high (+1 *SD*) levels of mindfulness.

**Figure 3:**
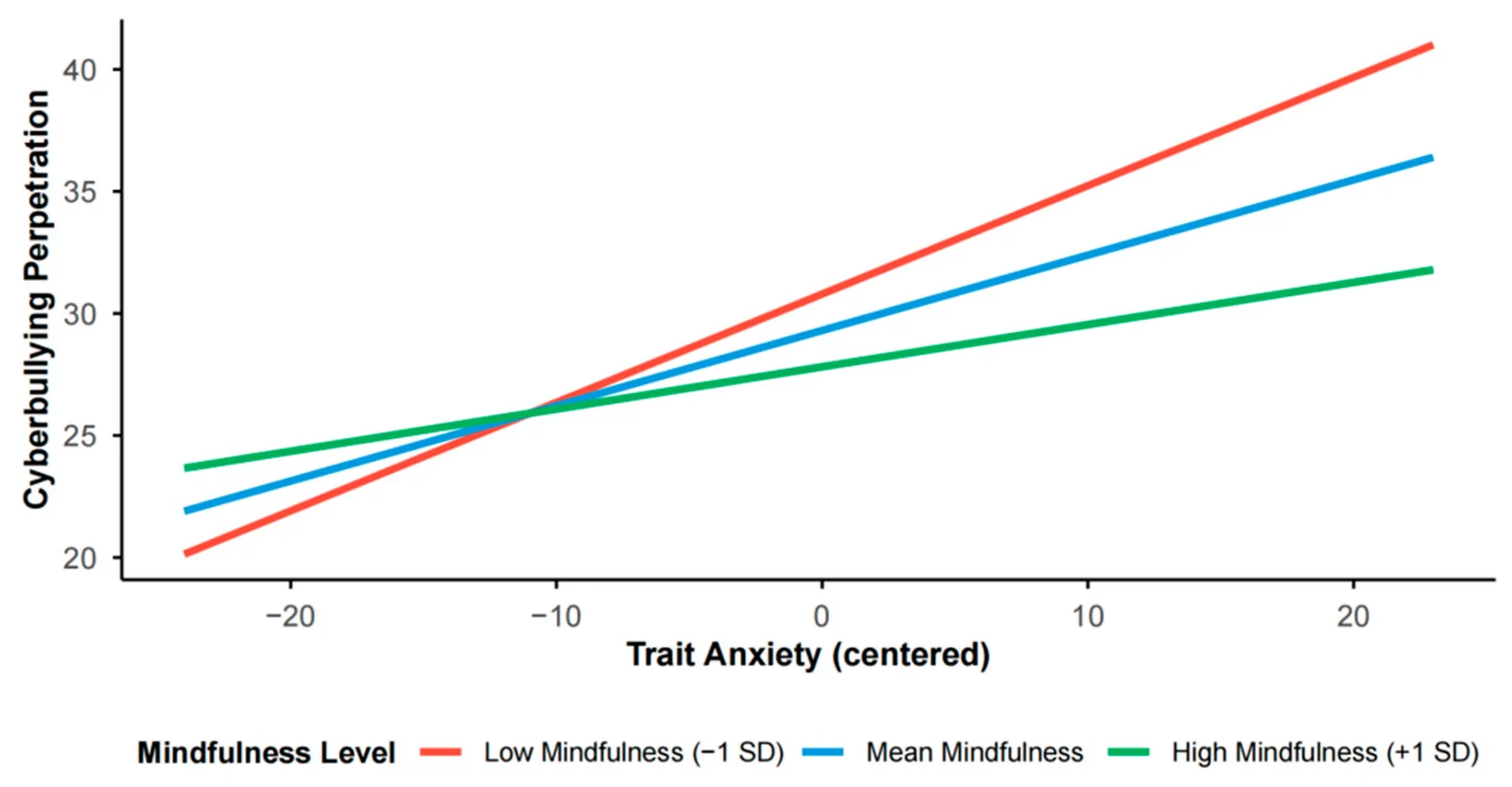
Interaction between trait anxiety and mindfulness on cyberbullying perpetration. Simple slopes are plotted at low (−1 *SD*), mean, and high (+1 *SD*) levels of mindfulness.

Simple slope analyses clarified these interactions: the positive association of trait anxiety with intolerance of uncertainty and cyberbullying weakened as mindfulness increased (Figure 2). Bootstrapped estimates (Table 4) showed that the indirect effect of trait anxiety on cyberbullying via intolerance of uncertainty was significant at all levels of mindfulness, though the effect attenuated as mindfulness increased (Figure 3). The index of moderated mediation was significant (Index = −0.030, 95% CI [−0.043, −0.018]), providing statistical evidence of a conditional indirect association consistent with the hypothesized model.

## 4. Discussion

The present study integrated trait anxiety, intolerance of uncertainty, mindfulness, and cyberbullying perpetration within a single conditional process model and tested this model in a large sample of Chinese university students. The results showed that trait anxiety was positively associated with cyberbullying and that intolerance of uncertainty served as a statistical intermediary in this association. Mindfulness moderated both the direct path and the first-stage path. These findings provide an integrated statistical account of how anxiety-related vulnerability, uncertainty-related cognition, and mindfulness-based regulation jointly relate to cyberbullying behavior. The contribution is primarily theory-consistent statistical integration and hypothesis generation for future longitudinal and intervention studies, rather than demonstration of a new causal mechanism.

The positive association between trait anxiety and cyberbullying is consistent with the GAM, which suggests that stable individual vulnerabilities may be associated with aggressive behavior through affective, cognitive, and arousal-based processes (Allen et al., 2018). Students with higher trait anxiety may be more sensitive to online ambiguity, interpersonal rejection, or perceived threat, making them more likely to respond defensively or aggressively in digital contexts (Sun et al., 2025). Because online communication often lacks clear social cues and may reduce immediate interpersonal accountability, anxious arousal may be more easily associated with maladaptive online behavior (Jeong et al., 2024). This interpretation aligns with prior work linking anxiety-related distress and digital aggression (Ramos et al., 2025; Yang et al., 2025).

Intolerance of uncertainty emerged as a significant statistical intermediary. This suggests that trait anxiety may be associated with cyberbullying partly through greater difficulty in tolerating uncertain or ambiguous social information. From a cognitive-behavioral perspective, individuals with high trait anxiety tend to perceive uncertainty as threatening and may seek rapid emotional relief or cognitive closure (Birrell et al., 2011; Wilson et al., 2026). In online settings, ambiguous posts, delayed replies, or unclear social feedback may heighten uncertainty-related distress. Cyberbullying may then be statistically associated with a maladaptive strategy for restoring a sense of control or reducing internal tension (Guo et al., 2025). Importantly, because the present study employed a cross-sectional design, all mediation findings should be interpreted as statistical associations that are consistent with the proposed theoretical model, rather than as evidence of causal ordering or temporal sequence. The indirect effect reported here represents a statistical pathway that is theoretically grounded but does not establish that trait anxiety precedes intolerance of uncertainty or cyberbullying in time.

Mindfulness weakened the positive association between trait anxiety and cyberbullying. This indicates that the link between anxiety proneness and online aggression was weaker among students who reported higher mindfulness (Zhu et al., 2025). Drawing on MAT, by promoting present-moment awareness and nonreactive acceptance, mindfulness may be associated with a lower likelihood that anxious thoughts and emotions are linked to aggressive behavior (Simione & Saldarini, 2023). Theoretically, this suggests that students with greater mindfulness may be better able to notice anxiety-related impulses, pause before responding, and choose less harmful ways of managing distress—although these intermediate processes were not directly measured in the present study. This interpretation is consistent with evidence that mindfulness is associated with stronger emotion regulation and cognitive flexibility (Kong et al., 2025; Pang et al., 2025; Raugh et al., 2025).

Mindfulness was associated with weaker statistical associations between trait anxiety and intolerance of uncertainty. This suggests that mindfulness may operate not only at the behavioral level but also at the cognitive-affective level (Hoge et al., 2021). When students are more mindful, trait anxiety may be less likely to be associated with rigid threat appraisal, excessive anticipation, or intolerance of uncertainty (Larochelle et al., 2025). In other words, mindfulness may help anxious individuals remain oriented toward present experience rather than becoming absorbed in uncertainty-related worry (Raugh et al., 2025; Sun et al., 2025). The decreasing conditional indirect effects across low, mean, and high mindfulness further support this interpretation.

The practical implications of these findings should be interpreted with caution given the modest effect sizes observed and the cross-sectional nature of the data. While the statistical significance of the moderated mediation model is robust due to the large sample size, the magnitude of the conditional indirect effects (ranging from 0.049 to 0.108) suggests that the practical significance of these effects is limited. Nevertheless, these findings suggest testable intervention hypotheses: if future longitudinal or experimental research confirms that trait anxiety is associated with cyberbullying through intolerance of uncertainty, then interventions designed to reduce anxiety reactivity and improve tolerance of uncertainty may have potential benefits for cyberbullying prevention. Similarly, the observed association between dispositional mindfulness and lower cyberbullying suggests—but does not demonstrate—that mindfulness training could reduce cyberbullying. Future research with longitudinal and intervention designs is needed before strong practical recommendations can be made. These constructs should be regarded as candidate targets for future intervention research, not as established intervention mechanisms.

## 5. Conclusions, Limitations, and Future Directions

The present study found that trait anxiety was positively associated with cyberbullying perpetration among university students and that intolerance of uncertainty served as a statistical intermediary in this association. Mindfulness moderated both the first-stage path and the direct path, such that the associations of trait anxiety with intolerance of uncertainty and cyberbullying were weaker among students with higher mindfulness. These findings highlight intolerance of uncertainty as a cognitive-affective statistical pathway linking anxiety-related vulnerability to cyberbullying and suggest that mindfulness may be a potential protective correlate in this process. The scientific contribution is incremental but meaningful: the study integrates these four constructs within one conditional process model and tests it in a large sample. Its value lies primarily in theory-consistent statistical integration and hypothesis generation for future longitudinal and intervention research, rather than in establishing causal mechanisms.

Several limitations should be noted. First, the cross-sectional design precludes definitive causal inference regarding the temporal sequence of trait anxiety, intolerance of uncertainty, and cyberbullying. Given the reciprocal nature of online aggression, where cyberbullying may exacerbate anxiety and uncertainty over time, future research should employ longitudinal designs to establish the prospective predictive power of these variables (Lin et al., 2025).

Second, the reliance on self-report measures may be constrained by social desirability bias, as cyberbullying often involves behaviors that individuals are reluctant to disclose due to their aggressive and stigmatized nature, likely leading to an underestimation of its true prevalence. Furthermore, self-reports may fail to capture the nuances of cyberbullying as a social process, particularly its covert or clandestine manifestations, which participants often strategically minimize to engage in impression management and mitigate potential social repercussions. To address this, future research should adopt a multi-method framework by triangulating self-reports with peer-nomination techniques or objective digital trace data, including social media interaction logs, to capture a more accurate and nuanced profile of aggressive tendencies in online settings. In addition, the single-source, single-occasion self-report design may raise concerns about common method variance. Although Harman’s single-factor test suggested that this was not a major threat (the first unrotated factor accounted for 16.85% of the total variance, below the 40% threshold), we acknowledge that Harman’s test has limited sensitivity and does not definitively exclude common-method variance (Podsakoff et al., 2012). Observed correlations should therefore be interpreted with appropriate caution and should not be assumed to represent entirely construct-specific relationships. Future research employing multi-method assessments (e.g., peer reports or objective digital trace data) would help to further mitigate this concern.

Third, although previous cybervictimization was measured and included as a covariate, the present study did not further examine the overlap between cybervictimization and cyberbullying perpetration. Treating cybervictimization only as a control variable may not fully capture its complex role, as it could plausibly function as an antecedent, a moderator, a reciprocal outcome, or part of a bully-victim process. This issue is particularly relevant because cyberbullying often occurs in reciprocal or repeated online interactions, and some students may be both victims and perpetrators. For individuals with high trait anxiety, previous cybervictimization may intensify threat appraisal, uncertainty-related distress, and defensive or retaliatory responses in online contexts. Therefore, treating cybervictimization only as a control variable may obscure its substantive role in the anxiety–uncertainty–cyberbullying pathway. Future studies should distinguish among pure perpetrators, pure victims, bully victims, and uninvolved students and examine whether cybervictimization functions as an antecedent, moderator, or part of a reciprocal process linking trait anxiety, intolerance of uncertainty, and cyberbullying.

Finally, because mindfulness was the central moderator in our conditional process model, the inability to empirically distinguish the five FFMQ facets in this sample is an important limitation. The five-correlated-factor model produced an improper solution (latent correlations 0.995–1.028; 8 of 10 ≥ 1.00), and the higher-order and ESEM models did not converge. Diagnostic checks ruled out scoring, coding, and polychoric correlation artifacts (maximum inter-item polychoric correlation = 0.381; all item–total correlations positive; all items within range), pointing instead to inadequate discriminant validity among the five facets (HTMT = 0.995–1.027; AVE = 0.291–0.320). An exploratory factor analysis with polychoric correlations further showed that only 20 of 39 items loaded ≥ 0.30 on any factor, and the five factors explained only 33.6% of the total variance. We therefore used the FFMQ total score only as a pragmatic manifest composite, and this decision does not imply that the FFMQ is psychometrically unidimensional. Future studies with samples showing clearer FFMQ factor separation should examine whether specific facets (e.g., nonjudging, nonreacting) play distinct moderating roles in the anxiety–uncertainty–cyberbullying pathway. In addition, the use of a convenience sample recruited from a single university in Hebei Province, China, limits the generalizability of the findings to other populations and cultural contexts. Future research should replicate these findings in more diverse samples across different regions and educational settings.

## Figures and Tables

**Table 1:** Confirmatory factor analysis fit indices for all scales.

Instrument	*χ* ^2^	*df*	CFI	TLI	RMSEA [90% CI]	SRMR
STAI-T	182.75	170	0.997	0.997	0.008 [0.000, 0.015]	0.025
IUS-12	65.04	54	0.999	0.999	0.013 [0.000, 0.023]	0.015
RCBI	78.02	77	1.000	1.000	0.003 [0.000, 0.016]	0.014
FFMQ (five correlated factors)	677.11	692	1.000	1.001	0.000 [0.000, 0.008]	0.021

Note. STAI-T = State-Trait Anxiety Inventory–Trait subscale; IUS-12 = Intolerance of Uncertainty Scale; RCBI = Revised Cyberbullying Inventory; FFMQ = Five Facet Mindfulness Questionnaire. All CFAs used the WLSMV estimator with items treated as ordered categories. For the FFMQ, the five-factor model produced latent correlations ≥ 1.00 (improper solution), and the higher-order and ESEM models did not converge. See Supplementary Materials Table S1 for full syntax and output.

**Table 2:** Descriptive Statistics and Correlation Analysis.

Variables	1	2	3	4
1. Trait Anxiety	1			
2. Intolerance of Uncertainty	0.38 ***	1		
3. Mindfulness	−0.25 ***	−0.22 ***	1	
4. Cyberbullying	0.42 ***	0.39 ***	−0.29 ***	1
M ± SD	45.01 ± 8.00	35.98 ± 8.95	117.00 ± 19.99	29.57 ± 8.25

Note. *** *p* < 0.001.

**Table 3:** Results of Regression Analysis for the Moderated Mediation Model.

Variables	Model 1 (Mediating Variable: IU)				Model 2 (Dependent Variable: CB)			
	*B*	*SE*	*β*	*p*	*B*	*SE*	*β*	*p*
Constant	39.115 ***	3.121		<0.001	16.780 ***	2.855		<0.001
TA (X)	0.391 ***	0.030	0.394 ***	<0.001	0.309 ***	0.027	0.300 ***	<0.001
IU					0.208 ***	0.025	0.226 ***	<0.001
MA (W)	0.065 ***	0.012	0.166 ***	<0.001	−0.075 ***	0.010	−0.182 ***	<0.001
X × W	−0.007 ***	0.001	−0.137 ***	<0.001	−0.007 ***	0.001	−0.125 ***	<0.001
Gender	0.045	0.461	0.003	0.922	1.095 **	0.397	0.066 **	0.006
Age	−0.121	0.145	−0.024	0.406	0.212	0.125	0.041	0.089
Grade	−0.220	0.213	−0.031	0.302	−0.044	0.183	−0.006	0.809
Major	0.305	0.466	0.019	0.512	0.180	0.401	0.011	0.653
Internet Usage Duration	−0.187	0.321	−0.017	0.560	0.107	0.276	0.009	0.698
Cybervictimization Experience	−0.774	0.482	−0.046	0.109	−0.158	0.415	−0.009	0.703
R^2^	0.186				0.291			
F	31.305			<0.001	50.831			<0.001

Note. ** *p* < 0.01, *** *p* < 0.001. TA = trait anxiety; IU = intolerance of uncertainty; MA = mindfulness; CB = cyberbullying perpetration. *B* represents unstandardized coefficients; *β* represents standardized coefficients. The total effect of TA on CB (not shown in the regression table) is *β* = 0.425 (*B* = 0.4386, *SE* = 0.0265, *p* < 0.001). Exact *p* values are reported for each individual coefficient to enhance transparency and reproducibility.

**Table 4:** Simple Slope Analyses for Interactions of Trait Anxiety × Mindfulness.

Outcome	Mindfulness Level	*B*	*SE*	*t*	*p*	95% CI
IU	Low (−1 *SD*)	0.482	0.045	10.71	<0.001	[0.394, 0.570]
IU	Mean	0.391	0.030	13.15	<0.001	[0.332, 0.450]
IU	High (+1 *SD*)	0.300	0.041	7.32	<0.001	[0.219, 0.381]
CB	Low (−1 *SD*)	0.398	0.039	10.21	<0.001	[0.321, 0.475]
CB	Mean	0.309	0.027	11.31	<0.001	[0.256, 0.362]
CB	High (+1 *SD*)	0.220	0.036	6.11	<0.001	[0.149, 0.291]

Note. Conditional slopes represent the association of trait anxiety with the respective outcome at each level of mindfulness. All confidence intervals were bias-corrected bootstrap 95% CIs (5000 resamples).

## Data Availability

The raw data supporting the conclusions of this article will be made available by the authors upon request.

## Supplementary Materials

The following supporting information can be downloaded at https://www.mdpi.com/article/10.3390/bs16101901/s1, Table S1: CFA fit indices for STAI-T, IUS-12, RCBI, and FFMQ; Table S2a: FFMQ measurement model diagnostics; Table S2b: Model comparison; Table S3: Comparison of the main model and the dummy-coded academic year model.
