# Supplementary material for: Trait Anxiety and Cyberbullying Among University Students: The Mediating Role of Intolerance of Uncertainty and the Moderating Effect of Mindfulness
Source: Behav Sci (Basel). 2026 Oct 14;16(10):1901. doi: 10.3390/bs16101901 (PMC13701519; doi:10.3390/bs16101901)
Supplement: Supplementary file 1 [file behavsci-16-01901-s001.zip › behavsci-4474159-supplementary.pdf]

Supplementary Materials

Table S1. CFA fit indices for STAI-T, IUS-12, RCBI, and FFMQ.

Scale	Model	χ^2	df	CFI	TLI	RMSEA [90% CI]	SRMR
STAI-T	Unidimensional	182.75	170	0.997	0.997	.008 [.000, .015]	.025
IUS-12	Unidimensional	65.04	54	0.999	0.999	.013 [.000, .023]	.015
RCBI	Unidimensional	78.02	77	1.000	1.000	.003 [.000, .016]	.014
FFMQ	Five correlated factors	677.11	692	1.000	1.001	.000 [.000, .008]	.021
FFMQ	Bifactor	632.71	663	1.000	1.001	.000 [.000, .006]	.020
FFMQ	Unidimensional	689.11	702	1.000	1.001	.000 [.000, .008]	.021

Note. Higher-order and ESEM models did not converge. Full lavaan syntax is provided in Supplementary S1.

Table S2a. FFMQ measurement model diagnostics.

Diagnostic	Result	Interpretation
Reverse-scoring check	All item–total correlations positive (0.503–0.580)	No scoring errors
Coding check	All items within 1–5, no missing values	No coding errors
Polychoric correlations (741 pairs)	Min = 0.234; Median = 0.308; Max = 0.381	No problematic polychoric correlations
Five-factor CFA latent correlations	0.995–1.028 (8/10 ≥ 1.00)	Improper solution
CFA standardized loadings	0.505–0.591 (all ps < .001)	Acceptable item-level loadings
Higher-order CFA	Did not converge	Non-positive definite latent covariance matrix
ESEM	Did not converge	Same reason
Bifactor CFA	χ^2 = 632.71, df = 663, CFI = 1.000, TLI = 1.001, RMSEA = .000, SRMR = .020	—
EFA (polychoric + promax)	Only 20/39 items ≥ .30; five factors explain 33.6% of variance	Five-facet structure not recovered
EFA factor correlations	0.870–0.905	Substantial overlap
HTMT	0.995–1.027 (8/10 ≥ 1.00)	Inadequate discriminant validity
AVE	0.291–0.320	Below 0.50 threshold
CR	0.739–0.756	Acceptable
Top modification indices	MF15 (MI = 251), MF1 (MI = 247), MF25 (MI = 237)	Local dependence

Note. STAI-T = State-Trait Anxiety Inventory–Trait subscale; RCBI = Revised Cyberbullying Inventory; IUS-12 = Intolerance of Uncertainty Scale; FFMQ = Five Facet Mindfulness Questionnaire. All CFAs used the WLSMV estimator with items treated as ordered categories.

The CFA models were re-run with lavaan 0.6-21 using WLSMV and ordered items. For the FFMQ, the five-factor model produced latent correlations ≥ 1.00 (improper solution), and the higher-order and ESEM models did not converge. See Supplementary Materials S1 for full syntax and output.

Table S2b. Model comparison.

Model	χ^2	df	CFI	TLI	RMSEA [90% CI]	SRMR	Convergence
Unidimensional	689.11	702	1.000	1.001	0.000 [.000, .008]	0.021	Converged
Five correlated factors	677.11	692	1.000	1.001	0.000 [.000, .008]	0.021	Converged (improper solution)
Higher-order	—	—	—	—	—	—	Did not converge
Bifactor	632.71	663	1.000	1.001	0.000 [.000, .006]	0.020	Converged
ESEM	—	—	—	—	—	—	Did not converge

Note. Higher-order and ESEM models did not converge due to non-positive definite latent covariance matrices, consistent with the improper five-factor solution

Table S3. Comparison of the main model and the dummy-coded academic year model.

Parameter	Main model (continuous grade)	Dummy-coded grade	Change
a1 (TA → IU)	B = 0.391, $p < .001$	B = 0.389, $p < .001$	−0.002
a2 (MF → IU)	B = −0.065, $p < .001$	B = −0.065, $p < .001$	0.000
a3 (TA×MF → IU)	B = −1.229, $p < .001$	B = −1.232, $p < .001$	−0.003
c1 (TA → CB)	B = 0.309, $p < .001$	B = 0.308, $p < .001$	−0.001
c2 (MF → CB)	B = −0.075, $p < .001$	B = −0.075, $p < .001$	0.000
c3 (TA×MF → CB)	B = −1.128, $p < .001$	B = −1.128, $p < .001$	0.000
b (IU → CB)	B = 0.207, $p < .001$	B = 0.207, $p < .001$	0.000
Indirect effect	B = 0.081, $p < .001$	B = 0.081, $p < .001$	0.000
Index of moderated mediation	B = −0.255, $p < .001$	B = −0.255, $p < .001$	0.000

Note. The dummy-coded model used first-year as the reference category and included three dummy variables (grade 2, grade 3, grade 4+). All coefficients remained significant and in the same direction; magnitudes changed only trivially (maximum absolute change = 0.003).
